# A Simple but Highly Effective Approach to Evaluate the Prognostic Performance of Gene Expression Signatures

**DOI:** 10.1371/journal.pone.0028320

**Published:** 2011-12-07

**Authors:** Maud H. W. Starmans, Glenn Fung, Harald Steck, Bradly G. Wouters, Philippe Lambin

**Affiliations:** 1 Department of Radiation Oncology (Maastro), GROW - School for Oncology and Developmental Biology, Maastricht University Medical Center, Maastricht, The Netherlands; 2 Department of CAD and Knowledge Solutions, Siemens Medical Solutions, Malvern, Pennsylvania, United States of America; 3 Ontario Cancer Institute/Princess Margaret Hospital, University Health Network, Toronto, Ontario, Canada; National Cancer Institute, United States of America

## Abstract

**Background:**

Highly parallel analysis of gene expression has recently been used to identify gene sets or ‘signatures’ to improve patient diagnosis and risk stratification. Once a signature is generated, traditional statistical testing is used to evaluate its prognostic performance. However, due to the dimensionality of microarrays, this can lead to false interpretation of these signatures.

**Principal Findings:**

A method was developed to test batches of a user-specified number of randomly chosen signatures in patient microarray datasets. The percentage of random generated signatures yielding prognostic value was assessed using ROC analysis by calculating the area under the curve (AUC) in six public available cancer patient microarray datasets. We found that a signature consisting of randomly selected genes has an average 10% chance of reaching significance when assessed in a single dataset, but can range from 1% to ∼40% depending on the dataset in question. Increasing the number of validation datasets markedly reduces this number.

**Conclusions:**

We have shown that the use of an arbitrary cut-off value for evaluation of signature significance is not suitable for this type of research, but should be defined for each dataset separately. Our method can be used to establish and evaluate signature performance of any derived gene signature in a dataset by comparing its performance to thousands of randomly generated signatures. It will be of most interest for cases where few data are available and testing in multiple datasets is limited.

## Introduction

In recent years, DNA microarray technology has been increasingly used in oncology. It has provided insight into the biological mechanisms underlying tumour formation and identified new therapy targets [1], [2]. However, most studies performed in this field identify gene sets, or so-called signatures, which can be used to improve diagnosis and risk stratification [3], [4], [5], [6]. These signatures can be acquired through supervised analysis methods [7]. Both patient microarray and clinical data are directly used to find the genes that correlate with tumour type or patient outcome [8], [9], [10], [11], [12]. Also biology-based signatures can be used for patient prognosis, which are usually derived from *in vitro* microarray data [2], [13], [14], [15]. Though the performance of these classifiers can be very high in the dataset studied, application of these signatures in other datasets is often limited and data reproduction is not straightforward [16]. Furthermore, signatures identified in comparable studies show little overlap in gene content [1], [10], [17], [18], [19], [20]. Michiels *et al.* [4] showed that identified gene lists were highly variable within one dataset and depended on the patients included in the training set. Further, they demonstrated that several published gene classifiers did not classify patients better than by chance. They stress that validation is an important issue in microarray research. Fan *et al.* [21] repeated and extended these analyses 5 years later and made similar conclusions. Moreover Boutros *et al.* [19] amongst other showed that the use of different statistical procedures could identify multiple highly prognostic signatures from one dataset [22], [23]. An extensive analysis of the effect of different statistics on ranked gene lists showed large variability [24].

A major challenge with DNA microarray technology is to take account of variability across a very large number of parameters [1]. This variability arises from several sources: the biological samples, hybridisation protocols, scanning, and image and statistical analysis [7]. In a recent review, Dupuy *et al.* [1] demonstrated that proper methodology in pre-processing and statistical analysis is essential in these sorts of studies. They found that a large subset of published microarray studies show flaws in the applied analysis; serious mistakes are made in the selection of genes and inadequate control of multiple testing is performed. The issue of multiple testing is crucial, as microarrays monitor the expression of thousands of genes, while the number of samples is relatively small.

Statistical significance of the differences in gene expression patterns for different patient groups or tumour types is often determined with traditional statistical testing procedures, such as the two-sample t-tests or Wilcoxon rank sum tests [1], [7], [20]. These procedures are challenged with serious multiplicity and without employment of a correction for multiple testing, the number of false positives will be extremely high. Various methods have been developed to overcome this problem of identifying differentially expressed genes and are used to create gene signatures [11], [19], [20], [25].

More importantly multiple testing is often not considered in evaluating the prognostic power of signatures. Once a signature is created, its prognostic power is determined with traditional survival statistics and standard cut-off values for significance. We hypothesise that this can lead to high numbers of false prognostic signatures when the number of evaluated datasets is limited. Therefore we sought to develop a simple method to take into account the high-dimensionality of microarrays in the phase of evaluating signature prognosticity.

To quantify the problem of multiple testing we have developed a method to test batches of random signatures in microarray datasets. We show that the average chance that a random signature produces a prognostic result in one dataset is approximately 10% but can range from 1% to ∼40%. Increasing the number of datasets reduces this false positive rate significantly. As a result of this high degree of variability amongst datasets, we developed a method that can be used to determine an appropriate threshold level of significance that must be reached for a given signature. This is done by testing a set of randomly chosen signatures along with the signature of interest within the dataset under investigation.

## Results

In order to assess the potential for identifying prognostic gene signatures by chance alone in microarray based datasets a method was developed to test the prognostic value of batches of randomly generated signatures. Six different publicly available microarray datasets with follow-up data were used (Table 1). These six datasets differ in number of patients, number of measured genes, number of reporters measured per gene, as well as platform and type of cancer.

**Table 1:** Overview of the analysed patient microarray datasets.

Dataset	Cancer type	Number of patients with survival data	Number of UnigeneIDs on array	Average number of reporters measured per UnigeneID	Source
**Miller**	Breast cancer	236	20,647	1.97	GEO accession GSE3494:http://www.ncbi.nlm.nih.gov/projects/geo/
**Wang**	Breast cancer	286	12,867	1.61	GEO accession GSE2034:http://www.ncbi.nlm.nih.gov/projects/geo/
**Van de Vijver**	Breast cancer	295	18,781	1.21	http://microarray-pubs.stanford.edu/wound_NKI/
**Zhao**	Renal cancer	177	5,640	1.40	SMD:http://smd.stanford.edu/
**Beer**	Lung cancer	86	5,396	1.15	http://dot.ped.med.umich.edu:2000/ourimage/pub/Lung/index.html
**Garber**	Lung cancer	24	4,936	1.15	SMD:http://smd.stanford.edu/

**Figure 1:**
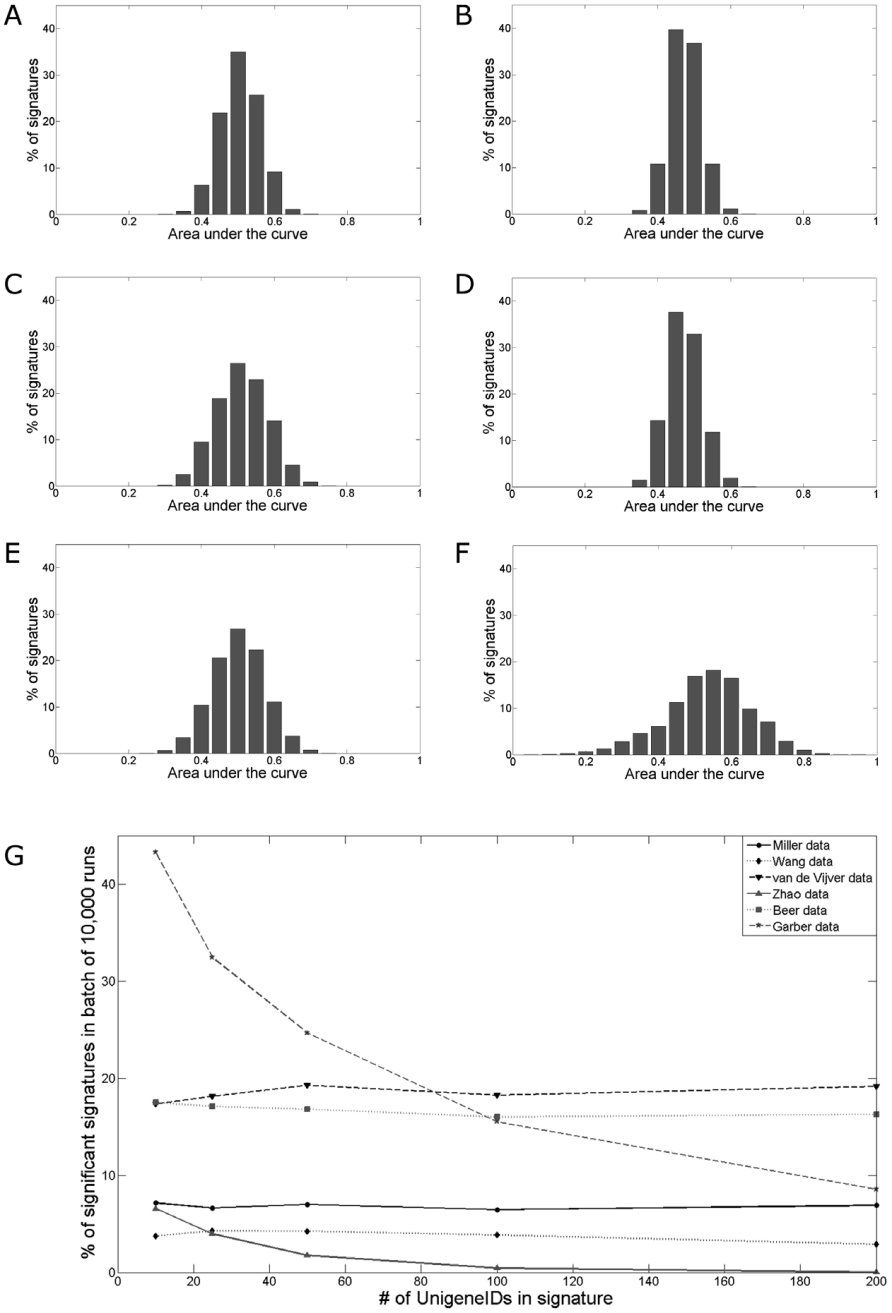
Random signature AUC distribution in different published microarray datasets. Batches of 10,000 random signatures were tested in six publicly available expression microarray datasets. Receiver-operator curves (ROC) were used to evaluate the signatures. The distribution of the AUC for the different datasets for the batch of 10,000 random signatures consisting of ten genes are displayed ((A) Miller dataset, (B) Wang dataset, (C) van de Vijver dataset, (D) Zhao dataset, (E) Beer dataset, (F) Garber dataset). The percentages of signatures that pass the criteria of AUC≤0.4 or AUC≥0.6 for the 5 batches of 10,000 runs are shown in (G).

For each dataset separately 5 batches of 10,000 random signatures were generated and tested. In each batch the number of genes (UnigeneIDs) in a gene set was predefined. The number of genes (UnigeneIDs) in the five batches were 10, 25, 50, 100 and 200 respectively. For example, the first batch included 10,000 random signatures, each consisting of ten genes. For each signature a patient score was derived, defined as the average of the expression of the genes in a signature (equation 1). Each signature score was then tested for prognostic value by ROC analysis and determination of the AUC. Figure 1A–F shows the distribution of the AUCs for the first batch of 10,000 random signatures for the different datasets.

To define a reasonable cut-off value for the AUC values, we first searched for AUCs used in published gene signatures. However, the majority of studies do not evaluate gene signatures using the AUC. Most gene signatures are evaluated with Kaplan-Meier survival curves and log-rank tests. Kaplan-Meier survival analyses and ROC analyses are linked; a high AUC corresponds to a good separation in distinct survival groups. To be able to define a cut-off, we calculated the AUCs for the different gene sets as evaluated in the review by Ntzani *et al.* [26]. The calculated AUCs as well as additional information are provided in Supplementary File S1. Based on these calculations we chose the cut-off values AUC≤0.4 and ≥0.6.

In Figure 1G the percentages of signatures that passed the criteria for the different batches of signatures are given. These percentages range from 1% to ∼40%, dependent on the dataset and the number of genes in the signatures. Table 2 provides the average, standard deviation as well as maximum and minimum AUC for the analyses with the gene sets consisting of ten genes. These data show that the larger the standard deviation, the higher the chance that a randomly generated signature is considered prognostic. Further, the maximum and minimum AUC show that very high signature performances can be found at random.

**Table 2:** A batch of 10,000 random signatures of 10 genes was tested in the six datasets.

Dataset	Average (± standard deviation) AUC	Maximum AUC	Minimum AUC
**Miller**	0.505±0.054	0.692	0.312
**Wang**	0.475±0.043	0.644	0.329
**van de Vijver**	0.499±0.073	0.744	0.297
**Zhao**	0.472±0.048	0.666	0.333
**Beer**	0.502±0.073	0.753	0.249
**Garber**	0.536±0.118	0.938	0.031

Sampling 10,000 gene sets is a small number compared to the total number of possible gene sets. In order to show that the 10,000 random gene sets are sufficient to estimate the AUC distribution, we tested batches of 1,000,000 signatures consisting of 10 genes in the six datasets. The AUC distributions for this permutation study were similar to the distributions for the batches of 10,000 gene sets (Table 3).

**Table 3:** A batch of 1,000,000 random signatures of 10 genes was tested in the six datasets.

Dataset	Average (± standard deviation) AUC	Maximum AUC	Minimum AUC
**Miller**	0.503±0.044	0.688	0.290
**Wang**	0.475±0.042	0.691	0.303
**van de Vijver**	0.511±0.072	0.778	0.209
**Zhao**	0.472±0.048	0.699	0.283
**Beer**	0.503±0.073	0.831	0.196
**Garber**	0.539±0.117	1.000	0.000

From the differences between the six datasets (Table 1), it could be that the number of patients, the number of genes (UnigeneIDs) and the number of reporters measured per gene influence the probability that a randomly chosen signature is considered prognostic. To further investigate the impact of these parameters, the Miller dataset was used. To determine the influence of patient number, the dataset was split in halves and in quarters. For these partial datasets the same five batches of 10,000 random signatures were tested. The influence of the number of genes was tested by splitting the dataset in half, this time based on genes rather than patients. Again, five batches of 10,000 random signatures were tested. To investigate the influence of the number of reporters measured per gene, again a set of five batches of 10,000 genes was tested on the dataset, considering only genes with more than one reporter. This was repeated, but for each gene only one reporter measurement was considered. Of these parameters only patient number influenced the false discovery rate. Results of the analysis to determine the influence of the number of genes and the number of reporters measured per gene are given in Supplementary File S1 and figures S1A and S1B.

### Influence of patient numbers

**Figure 2:**
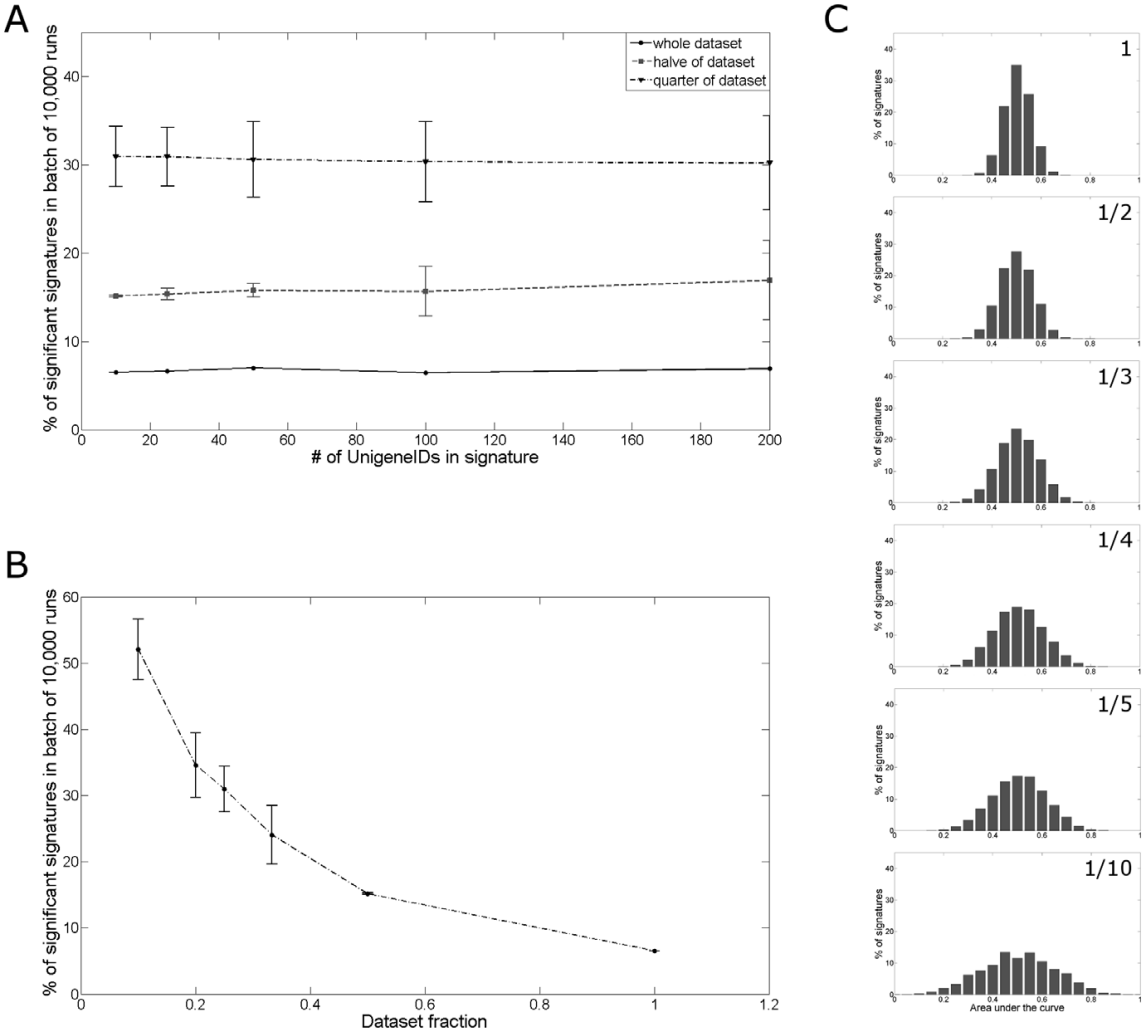
Effect of dataset size on random signature AUC distribution. Percentages of signatures that pass the criteria of AUC≤0.4 or AUC≥0.6 for the 5 batches of 10,000 runs for the Miller dataset, groups consisting of half the Miller dataset patients and groups with a quarter of the Miller dataset patients (**A**). Distribution of the AUC for the different subdivisions of the Miller dataset, for the batch with 10,000 random signatures consisting of ten genes (**B**). Relationship between size of the dataset and the false discovery rate in the Miller dataset (**C**).

It has already been reported in previous studies [26], [27] that the number of patients influences the false discovery rate. The Miller dataset was split into two and four groups respectively to confirm the importance of this factor. The same 5 batches of 10,000 random signatures that were tested on the whole dataset were tested on these subgroups (Figure 2A). Indeed the number of prognostic signatures increases dramatically when the size of the patient group decreases. To characterize the relationship between patient number and the probability that a randomly chosen signature is considered prognostic, additional analyses were performed for the batch of 10,000 runs with ten genes. The dataset was split into three, five and ten groups respectively. Figure 2B shows the distribution of the AUCs for the batch of 10,000 random signatures consisting of ten genes for the different dataset sizes. It is clear that the smaller the dataset, the wider and flatter the distribution becomes. Figure 2C presents the number of prognostic signatures as a function of dataset size.

### Effect of filtering

One of the parameters that could account for differences in the number of prognostic signatures for a given dataset is filtering. To briefly explore the influence of filtering, two simple filtering methods were applied on the Miller dataset. After this filtering, again five batches of 10,000 signatures were tested.

The first filtering procedure was to only consider reporters that had no absent calls in the patients. This very stringent filtering resulted in a reduction in number of reporters from ∼45,000 to ∼7,300 (approximately 5,000 unique UnigeneIDs). The second filtering method, often used in microarray based studies, consists of simply applying a threshold to the fold change. To show the effect of this step on the number of false positives a twofold threshold was applied. Only genes that show at least a two-fold change across the patients are considered. This reduced the number of reporters from ∼45,000 to ∼23,000.

The results for these analyses show that both filtering methods have a different effect (Figure S1C). Fold change filtering did not influence the probability that a randomly chosen signature is considered prognostic; rather, it provides similar results to those of non-filtered analysis. Filtering for absent reporters, on the other hand, introduced a signature size dependency for the false positive rate. A small signature size resulted in a false positive rate of ∼10%, whereas large signatures had a false discovery rate of only ∼0.5%. The average, however, stands at 5–6%, similar to the non-filtering and fold change filtering analyses.

### Signature testing procedure

To demonstrate that this random signature method can be used with all sorts of signature evaluation methods, two additional evaluation procedures were tested in the Miller dataset. In the previous analyses the signature score was used as continuous variable.

Here we selected 10,000 random samples of 10, 25, 50, 100 and 200 genes. In the first setup the signature score was used to median dichotomize the patients. In the second setup these gene subsets were in a K-nearest neighbor classification (KNN) combined with leave-one-out-cross validation (LOOCV). Both procedures results in patient classification into two groups, which were then coupled to outcome and evaluated by the AUC. Similar AUC distributions are obtained with these different signature evaluation procedures, exact distributions characteristics differ slightly (Table 4). The numbers of random gene sets passing the criteria are comparable (Figure S1D).

**Table 4:** Batches of 1,000,000 random signatures of 10, 25, 50, 100 and 200 genes were tested in the Miller dataset, where three different signature evaluation procedures were used.

	Signature score	Median dichotomized	KNN with LOOCV
% UnigeneIDs in signature	Average AUC(± standard deviation) [min - max]	Average AUC(± standard deviation) [min - max]	Average AUC(± standard deviation) [min - max]
**10**	0.505±0.054	0.535±0.045	0.553±0.036
	0.312–0.692	0.348–0.702	0.426–0.717
**25**	0.507±0.054	0.535±0.045	0.558±0.033
	0.313–0.692	0.338–0.690	0.412–0.697
**50**	0.509±0.055	0.538±0.045	0.561±0.031
	0.304–0.694	0.374–0.671	0.457–0.678
**100**	0.513±0.053	0.540±0.044	0.562±0.030
	0.319–0.682	0.352–0.679	0.435–0.689
**200**	0.518±0.052	0.543±0.043	0.562±0.029
	0.335–0.684	0.373–0.688	0.449–0.691

### Evaluating signatures by random testing

**Figure 3:**
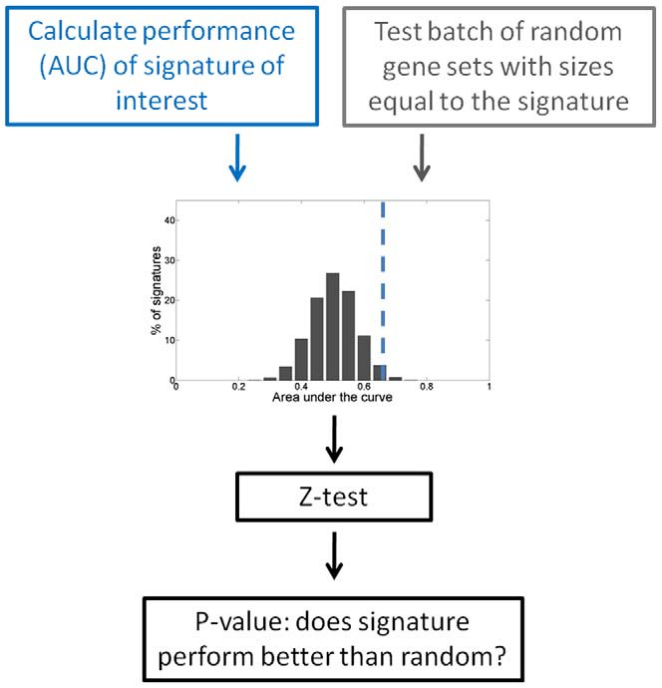
Workflow signature testing procedure. A systematic overview of the proposed signature testing procedure is depicted here. First performance of the signature of interest is determined. A batch of random gene sets with the same size as the signature is subsequently tested. Signature performance is then compared to the AUC distribution of the random gene sets with a Z-test to address whether the signature performs better than random.

To show that the random signature testing method is a valuable tool in microarray based studies, several published gene signatures were tested. In short, the suggested procedure to test a signature in a dataset is as follows (Figure 3). For the signature of interest the AUC was calculated in the dataset, additionally the AUC distribution for batches of random signatures with a similar size as the signature of interest was computed. The signature AUC was then compared to the random signature AUC distribution with a Z-test to assess whether the signature of interest performed better than could be expected by chance.

The Wound signature [13], “invasiveness gene signature” (IGS) [10] and two early hypoxia signatures [15] are recently published gene signatures. For the Wound and IGS signatures it was previously shown that these signatures had high prognostic value in different datasets and cancer types [10], [13], [28]. The two early hypoxia signatures however, were only evaluated in one dataset [15]. These signatures were evaluated in the three breast cancer datasets [8], [29], [30] with the signature score (details are provided in Supplementary File S1).

For the Miller dataset also Kaplan-Meier survival analyses were performed, since the two early hypoxia signatures were previously tested in this dataset. The results of Kaplan-Meier survival analyses and the random signature testing are given in Table 5 and Figure S3. From the Kaplan-Meier survival analyses all four signatures seemed to have a high prognostic value (p-values log-rank test <0.05). However the random signature testing procedure indicated that the two early hypoxia signatures did not perform better than chance in that dataset. Testing the four signatures in the other two breast cancer datasets indeed showed that the two early hypoxia signatures did not have prognostic value (p-values log-rank test >0.05). For the Wound and IGS signatures both evaluation procedures indicated that the performance of these signatures is high in the different datasets and that this is unlikely due to chance.

**Table 5:** Evaluating 4 published signatures with the random signature testing procedure in three breast cancer datasets.

	Wound signature				
Dataset	p-value [log rank test]	HR [95% CI]	p-value [Wald test]	AUC	p-value [Z-test]
**Miller**	0.001	2.48 [1.40–4.41]	0.002	0.671	0.002
**Wang**	0.016	1.59 [1.08–2.36]	0.017	0.597	0.001
**van de Vijver**	7.27 10^−9^	4.15 [2.42–7.04]	7.71 10^−8^	0.688	0.018

	IGS signature				
Dataset	p-value [log rank test]	HR [95% CI]	p-value [Wald test]	AUC	p-value [Z-test]
**Miller**	5.28 10^−4^	2.66 [1.49–4.78]	7.93 10^−4^	0.645	0.010
**Wang**	8.11 10^−5^	2.17 [1.45–3.25]	1.17 10^−4^	0.644	5.80 10^−5^
**van de Vijver**	2.16 10^−6^	3.12 [1.88–5.15]	6.35 10^−6^	0.668	0.038

	Early 0%				
Dataset	p-value [log rank test]	HR [95% CI]	p-value [Wald test]	AUC	p-value [Z-test]
**Miller**	0.014	2.00 [1.13–3.53]	0.015	0.601	0.083
**Wang**	0.051	0.69 [0.47–1.02]	0.055	0.452	0.001
**van de Vijver**	0.786	1.06 [0.67–1.67]	0.785	0.508	0.162

	Early 2%				
Dataset	p-value [log rank test]	HR [95% CI]	p-value [Wald test]	AUC	p-value [Z-test]
**Miller**	0.026	1.85 [1.06–3.22]	0.027	0.593	0.105
**Wang**	0.545	0.89 [0.61–1.32]	0.558	0.498	0.045
**van de Vijver**	0.321	1.25 [0.80–1.97]	0.321	0.549	0.418

## Discussion

We assessed six patient microarray datasets spanning different cancer types, numbers of patients and arrays to evaluate the effect of false positives on gene signature evaluation. Different-sized batches of 10,000 random signatures were tested in all datasets. With the given threshold, the average chance that a randomly generated signature was considered prognostic was approximately 10%, but ranged from 1% to ∼40%.

Testing batches of random signatures in different datasets revealed that the AUC distribution varied widely between datasets. Choosing an arbitrary cut-off value for significance is then clearly not suited for gene signature evaluation. Rather a dataset-based cut-off value should be considered. The random testing method we propose here can be applied to calculate the level of AUC necessary to reach significance beyond random for a given signature size in a given dataset.

The random testing procedure can also be used to directly test whether the performance of a certain signature could be due to chance. A schematic overview is given in Figure 3. A batch of random signatures with the same size as the signature of interest can be tested along with the original signature. The AUC distribution of the random signatures can then be used to statistically test whether the original signature performs better than random. An equivalent permutation-based validation step was used by Boutros *et al.* [19] to evaluate their signature; this step provided significant information on the prognostic performance of the gene set.

We have shown that proper validation is absolutely essential in gene signature research. This supports several previous studies, which have argued that signature performance is often overestimated due to improper validation in a large number of studies [1], [26]. For several analyses, the maximum and minimum AUC were also calculated. We show that random signatures can have very high performances (AUC>0.9), which further supports this observation.

A method to overcome this multiple testing problem is validation in multiple independent datasets. We have shown that testing random signatures in two datasets decreased the chance that a random signature is called prognostic dramatically (Figure S2). However it is not always possible to validate a gene signature in multiple datasets. In oncology most microarray studies focus on breast and lung cancer, for these sites there are a lot of public datasets available that can be used for validation. Therefore this technique is not primarily meant for these cancer types, but rather for tumour types where only few data, in terms of the number of samples and number of datasets, are available; for those cases this technique would be valuable. By comparing the performance of four published signatures in one patient microarray dataset with Kaplan-Meier curves all signatures seemed to have prognostic value. However, applying the random testing procedure in that dataset already indicated that two out of four signatures did not perform better than chance. Testing the four signatures in multiple datasets indeed showed that these two signatures did not show prognostic value in the other datasets.

From the analyses on all six datasets, several parameters could influence the number of false positives. To assess the effect of these variables, several parameters were manipulated in one of the datasets. However, of the tested parameters, only patient number influenced the false positive rate dramatically. The need for large patient groups to obtain reliable results has already been recognised in other studies. Ntzani *et al.* [26] evaluated 84 microarray studies and concluded that small studies often give inflated, over-promising results. Zien *et al.* [27] assessed the influence of the number of samples in a different way: a simulation model was applied in which specificity and sensitivity were measured depending on changes in sample size, technical and biological variability. They showed that with small sample sizes, sensitivity and specificity were highly dependent on the biological and technical variance, whereas larger sample sizes led to quite robust results that were less dependent on biological and technical variance. Moreover Popovici *et al.* [23] tested the effect of training set size on the performance of the trained marker in a validation dataset. Overall signature performance improved in the validation data and better concordance between training and testing results was observed when training dataset size increased.

Testing batches of random generated gene sets in different gene expression microarray datasets showed that the use of an arbitrary cut-off value for evaluation of signature significance is not suitable. Further it is important to use the same signature evaluation procedure for the random gene sets as for the signature of interest, since the AUC distribution can differ when using a different method. Thresholds should be defined for single datasets separately in order to obtain reproducible results. This permutation method can be used to establish and evaluate signature performance of any derived gene set within single or multiple datasets by comparing its performance to the performance distribution of thousands of randomly generated signatures. However it will be of most interest for cases where limited data is available.

## Methods

### Random signature testing

A method to test the prognostic value of random gene signatures of a predefined size on a microarray dataset was developed in Matlab (Matlab 7.1, The Mathworks, Natick, MA, USA). Unless indicated otherwise, analyses were performed using this program. The program creates a user-specified number of random gene sets, consisting of a user-specified number of genes. For a given dataset all genes on the respective microarray were used to create the random signatures. This batch of random signatures was then tested on a dataset by means of a signature score calculation.

### Datasets

Patient microarray and clinical follow-up data were collated to test the random gene sets. Datasets are publicly available in the microarray databases Gene Expression Omnibus (GEO: http://www.ncbi.nlm.nih.gov/projects/geo/) and Stanford Microarray Database (SMD: http://genome-www.stanford.edu/microarray) and elsewhere. Accessory clinical and follow-up data were also given or provided by the authors on request. Table 1 provides an overview of the datasets and the databases, where these are accessible. Data filtering and pre-processing is explained in Supplementary File S1.

#### Signature score calculation

Expression data of the genes in a signature was extracted from the dataset. The following step was used to calculate a signature score for each patient included in the dataset. This score was defined as the average expression value of the genes in the signature (equation (1)). When a gene was represented by more than one reporter on an array, the expression of the reporters was averaged before signature calculation. The signature scores for each patient were then coupled to the survival data of the patients.
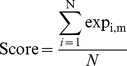
(1)


Where: Score, signature score; N, number of genes in the signature; exp_i,m_, gene expression of gene i in sample m.

The signature score was used to median dichotomize the patient cohorts.

In a second setup expression of the genes in the signature were used for K-nearest neighbor classification (KNN) combined with leave-one-out-cross validation (LOOCV). With this method one patient is withheld and the class membership of this patient is predicted using the KNN model (knnclassify function in Matlab) built on the remaining patients. The event parameter of the survival data was used as training class. This procedure was repeated for each patient, resulting in a class prediction for the whole cohort.

### Analysis

The signature scores, median dichotomized groups or KNN classifications were evaluated with the area under the curve (AUC) of the receiver operator curve (ROC). Definitions for AUC calculations are as follows:

True positive: patient in the high score group that died from disease
False positive: patient in the high score group that is alive
True negative: patient in the low score group that is alive
False negative: patient in the low score group that died from disease

A signature score was considered prognostic when the AUC is ≤0.4 or ≥0.6. This cut-off value was based on the AUCs of several published gene signatures evaluated in the study of Ntzani *et al.* [26] (further details are given in the results section and Supplementary File S1).

## Supporting Information

Figure S1 **Effect of number of genes, number of probes per gene, filtering and signature evaluation procedure on AUC distribution.** A: Percentages of signatures that pass the criteria of AUC≤0.4 or AUC≥0.6 for the 5 batches of 10,000 runs for the Miller dataset, groups consisting of half of the UnigeneIDs of the Miller dataset. B: Percentages of signatures that pass the criteria of AUC≤0.4 or AUC≥0.6 for the 5 batches of 10,000 runs for the Miller dataset, taking only one or multiple reporters per gene into account. C: Percentages of signatures that pass the criteria of AUC≤0.4 or AUC≥0.6 for the 5 batches of 10,000 runs for the Miller dataset, taking two different filtering methods. D: Percentages of signatures that pass the criteria of AUC≤0.4 or AUC≥0.6 for the 5 batches of 10,000 runs for the Miller dataset, taking three different signature evaluation procedures (KNN LOOCV: K-nearest neighbor classification with leave-one-out cross validation).
(TIF)

Figure S2 **Evaluating random gene sets in multiple datasets.** Percentages of signatures that pass the criteria of AUC≤0.4 or AUC≥0.6 for the 5 batches of 10,000 runs for the van de Vijver and Beer datasets separately and combined.
(TIF)

Figure S3 **Kaplan-Meier survival analysis of 4 published gene signatures.** Kaplan-Meier survival curves for the Miller dataset for 4 published signatures (A: Wound signature, B: IGS, C: early hypoxia 0% and D: early hypoxia 2%).
(TIF)

File S1 The supplementary material contains a section with supplementary materials and methods and a section supplementary results. The supplementary materials and methods is a more detailed description of the data analyses. The supplementary results describe the analyses to check the influence of several parameters on the random signature AUC distribution that had minimal to no effect. Further additional tables are included.
(DOC)
