# Supplementary figures and images for: A Simple but Highly Effective Approach to Evaluate the Prognostic Performance of Gene Expression Signatures
Source: PLoS One. 2011 Dec 7;6(12):e28320. doi: 10.1371/journal.pone.0028320 (PMC3233554; doi:10.1371/journal.pone.0028320)

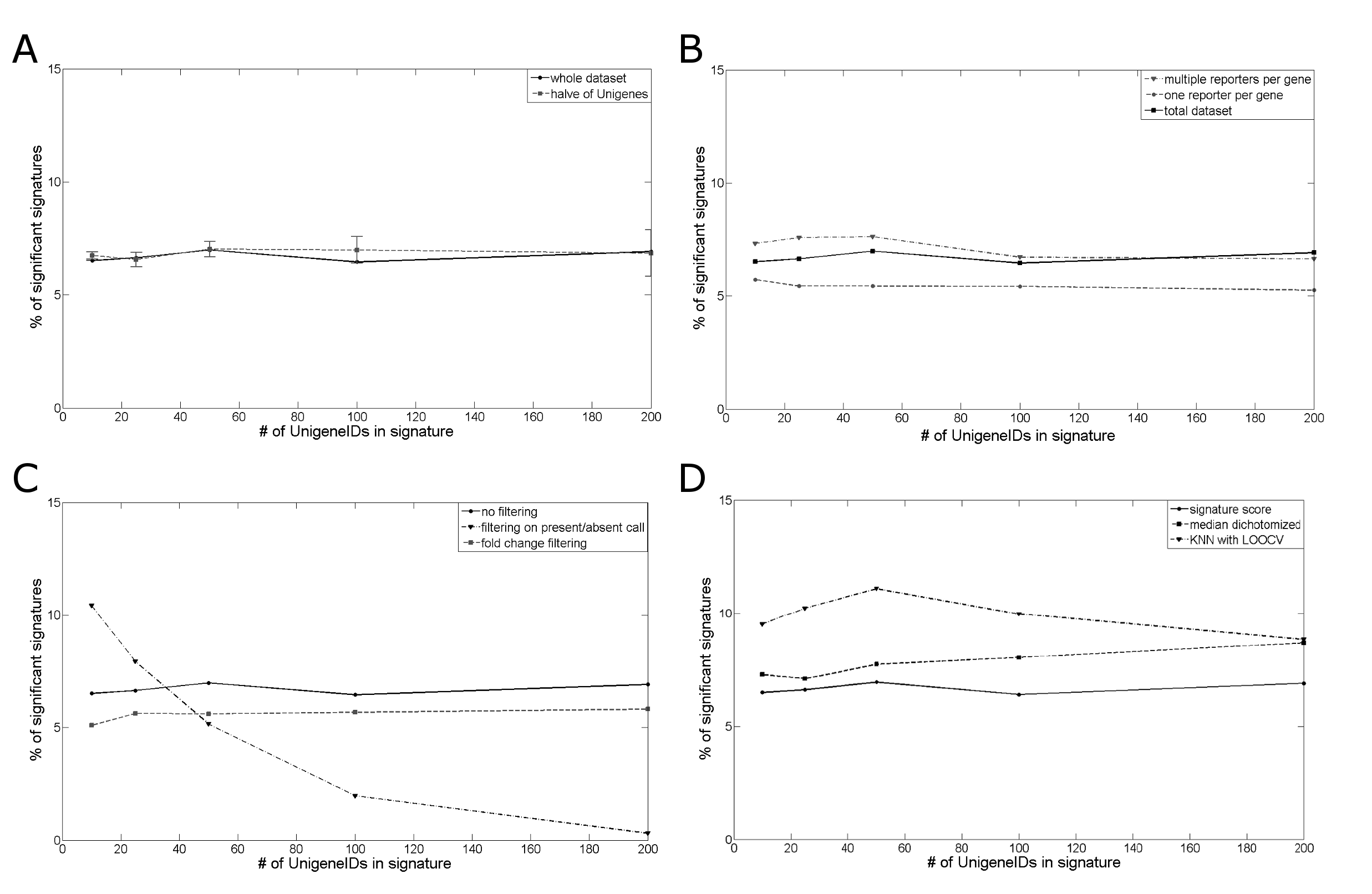

Supplement: Figure S1 — Effect of number of genes, number of probes per gene, filtering and signature evaluation procedure on AUC distribution. A: Percentages of signatures that pass the criteria of AUC≤0.4 or AUC≥0.6 for the 5 batches of 10,000 runs for the Miller dataset, groups consisting of half of the UnigeneIDs of the Miller dataset. B: Percentages of signatures that pass the criteria of AUC≤0.4 or AUC≥0.6 for the 5 batches of 10,000 runs for the Miller dataset, taking only one or multiple reporters per gene into account. C: Percentages of signatures that pass the criteria of AUC≤0.4 or AUC≥0.6 for the 5 batches of 10,000 runs for the Miller dataset, taking two different filtering methods. D: Percentages of signatures that pass the criteria of AUC≤0.4 or AUC≥0.6 for the 5 batches of 10,000 runs for the Miller dataset, taking three different signature evaluation procedures (KNN LOOCV: K-nearest neighbor classification with leave-one-out cross validation). (TIF) [file pone.0028320.s001.tif]

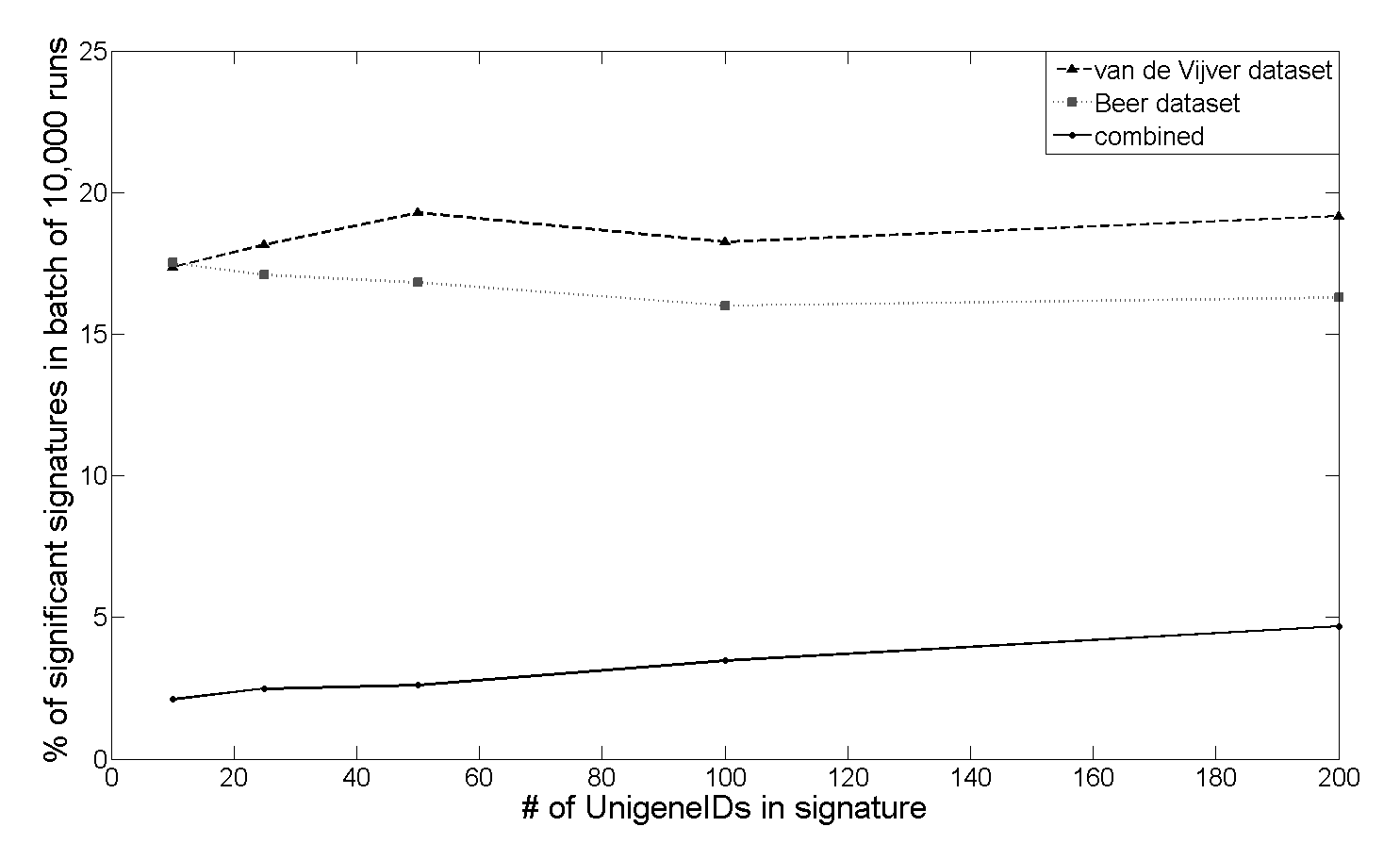

Supplement: Figure S2 — Evaluating random gene sets in multiple datasets. Percentages of signatures that pass the criteria of AUC≤0.4 or AUC≥0.6 for the 5 batches of 10,000 runs for the van de Vijver and Beer datasets separately and combined. (TIF) [file pone.0028320.s002.tif]

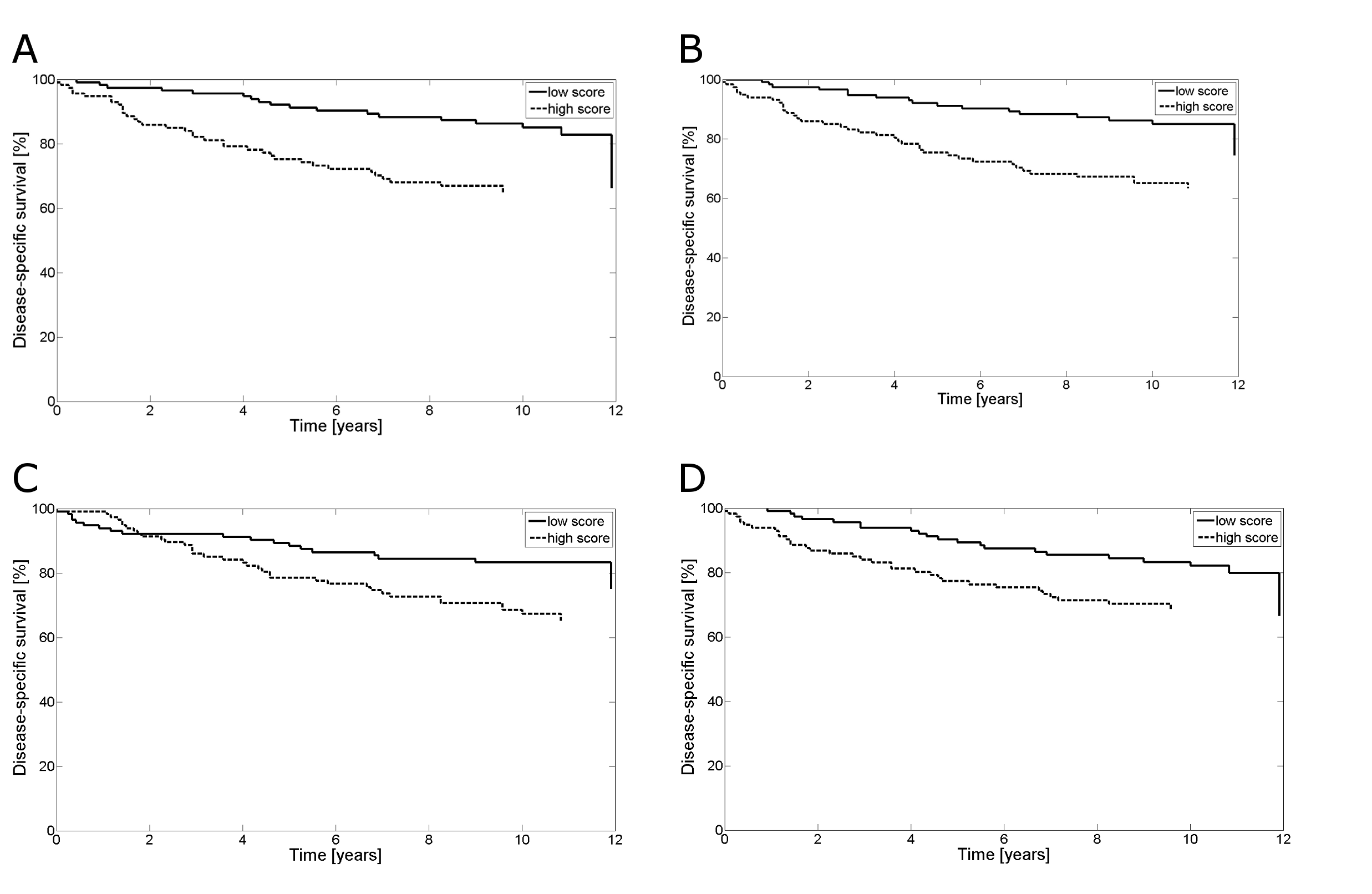

Supplement: Figure S3 — Kaplan-Meier survival analysis of 4 published gene signatures. Kaplan-Meier survival curves for the Miller dataset for 4 published signatures (A: Wound signature, B: IGS, C: early hypoxia 0% and D: early hypoxia 2%). (TIF) [file pone.0028320.s003.tif]
