# Supplementary material for: A Simple but Highly Effective Approach to Evaluate the Prognostic Performance of Gene Expression Signatures
Source: PLoS One. 2011 Dec 7;6(12):e28320. doi: 10.1371/journal.pone.0028320 (PMC3233554; doi:10.1371/journal.pone.0028320)
Supplement: File S1 — The supplementary material contains a section with supplementary materials and methods and a section supplementary results. The supplementary materials and methods is a more detailed description of the data analyses. The supplementary results describe the analyses to check the influence of several parameters on the random signature AUC distribution that had minimal to no effect. Further additional tables are included. (DOC) [file pone.0028320.s004.doc]

**Supplementary File S1**

**Supplementary materials and methods**

*Data filtering and pre-processing*

Datasets downloaded from the SMD [1, 2] were filtered according to the parameters in the paper. CloneIDs were chosen as gene annotation and the data obtained was log-transformed. For the normalized affymetrix arrays [3, 4] the genes were log-transformed. The Beer *et al*. [5] dataset was already pre-processed therefore to perform log-transformation all expression values below 1.1 were set to 1.1, this was similar to the processing performed by Chen *et al.*[6]. In all other cases the data were kept in downloaded, log-transformed format [7]. CloneIDs and affymetrix probeIDs were translated into UnigeneIDs (Build199) with Source (http://smd.stanford.edu/) or Affymetrix data files (Affx annotation files available at www.affymetrix.com). Datasets were imported in Matlab.

*AUC cut-off value definition*

Ntzani *et al*. [8] evaluated the performance of different gene sets by means of sensitivity and specificity. These parameters were used to calculate the AUC. In their study they evaluated the performance of gene sets during cross-validation (Figure 1 upper panel in Ntzani *et al*. [8]), on independent validation (Figure 1 lower panel in Ntzani *et al*. [8]) and based on unsupervised approaches (Figure 2 in Ntzani *et al*. [8]).

For each of the three figures we calculated the AUC for each data point. In Supplementary Table S1 the sensitivity, specificity, AUC and dataset size are provided for the three figures. From these data it is obvious that during cross-validation the performance of gene sets is high, as expected. Further it is clear that the performance of gene sets is better when datasets are small.

In Supplementary Table S2 the average AUC is calculated for the three figures. The use of small datasets often leads to overestimated results [8, 9]; thus, we also calculated the average AUC by omitting datasets smaller than 10, 20, 30, 40 and 50 patients respectively.

To define a threshold for AUC, not all data are included. During cross-validation the performance of a gene set is optimized and high values for AUC are found; therefore, these are omitted. Further, in our study five out of six datasets included >> 50 patients. Based on these calculations a threshold of ≤0.4 or ≥0.6 was chosen.

*Evaluation of published signatures*

The four published signatures evaluated here [10-12] consist of gene that are both upregulated and downregulated in a certain phenotype. Therefore the definition of the score was slightly different form the score used for the random batches of signatures; a weighted average was used (equation 2). For the Wound signature weights of -1 and 1 were assigned to genes representing a quiescent and activated wound response respectively. For the IGS signature log ratios were provided in the supplementary data of the paper, a weight of 1 is assigned to genes with a positive log ratio and a weight of -1 to genes with a negative log ratio. The two early hypoxia signatures contained only genes that are upregulated early in hypoxia, therefore all weights were 1.

$\text{Signature score }=\text{ }\frac{\sum_{i=1}^{\text{N}} \text{weight}_{\text{i}}\cdot\text{expression of gene}_{\text{i,m}}}{N}$ equation 2

Where: N: the number of genes in the signature

Patients were subsequently grouped into a high and a low risk group for each signature by median dichotomization of the signature scores. Kaplan-Meier survival curves with log-rank tests and Cox proportional hazard ratio modelling were performed to address survival differences between the two groups.

**Supplementary results**

*Influence of number of genes*

The number of genes (UnigeneIDs) might influence the probability that a randomly chosen signatures is considered prognostic. To test this, the Miller dataset was used again. This time the dataset was split in two based on the genes, and five batches of 10,000 random signatures were tested on each part. Supplementary Figure 1A shows that the percentage of prognostic signatures is not influenced by the number of genes present on the microarray. To ascertain that the influence of the number of genes is negligible the dataset was split in ten, based on the genes. A run of 10,000 random signatures consisting of ten genes was performed. This showed that splitting the dataset into more groups based on the number of genes did not result in a change in the false positive rate. The average chance of finding a prognostic result at random was 6.9% ± 0.8 for the ten groups, similar to the number found for the whole dataset.

*Influence of the number of reporters measured per gene*

Also the number of reporters measured per gene could influence the probability that a randomly chosen signatures is considered prognostic. To test this hypothesis only the genes represented by more than one reporter on the Miller dataset were taken into account. The Miller dataset has approximately 10,000 UnigeneIDs (genes) that are represented by multiple gene identifiers (probes in the microarray).

First, five batches of 10,000 random signatures, consisting only of the genes mentioned, were tested. In the second run of five batches, only one reporter per gene was taken into account from this list. If the number of reporters per gene plays a role, considering only one rather than multiple reporters was thought to increase the change that a randomly chosen signature was considered prognostic. The results given in Supplementary Figure 1B show that the number of reporters per gene did not influence the false positive rate.

*Validation in multiple datasets*

To show that the probability that a randomly chosen signatures is considered prognostic will substantially decrease when random signatures are tested in two independent datasets, five batches of 10,000 random signature runs were once more performed, however now the batches of signatures were tested on two datasets in parallel. It is clear that the van de Vijver and Beer dataset had the highest number of false positives overall, therefore these datasets were analyzed in parallel. The results of the 10,000 runs on the two datasets, both separately and combined (presented in Supplementary Figure 2), show that the chance of finding a prognostic result at random dropped from ≥15% to ≤5% by combining the two datasets. Addition of another independent dataset will further decrease this number.

Supplementary tables

| Table S1. Calculated AUC for the gene sets evaluated in the review by Ntzani *et al*. [8] | | | | |
| --- | --- | --- | --- | --- |
|  | | | | |
| **Figure 1 upper panel Ntzani *et al*. [8]** | | | | |
| **Dataset** | **Sensitivity** | **Specificity** | **AUC** | **Dataset size** |
| 1 | 45 | 95 | 0.70 | 60 |
| 2 | 90 | 70 | 0.80 | 78 |
| 3 | 100 | 100 | 1 | 10 |
| 4 | 65 | 65 | 0.65 | 86 |
| 5 | 100 | 90 | 0.95 | 29 |
| 6 | 70 | 70 | 0.70 | 31 |
| 7 | 55 | 85 | 0.70 | 58 |
| 8 | 100 | 100 | 1 | 33 |
| 9 | 85 | 85 | 0.85 | 20 |
|  | | | | |
| **Figure 1 lower panel Ntzani *et al*. [8]** | | | | |
| **Dataset** | **Sensitivity** | **Specificity** | **AUC** | **Dataset size** |
| 1 | 100 | 70 | 0.85 | 19 |
| 2 | 90 | 50 | 0.70 | 180 |
| 3 | 60 | 65 | 0.625 | 43 |
| 4 | 45 | 65 | 0.55 | 64 |
| 5 | 40 | 55 | 0.475 | 58 |
| 6 | 70 | 65 | 0.675 | 80 |
| 7 | 85 | 90 | 0.875 | 27 |
| 8 | 75 | 100 | 0.875 | 6 |
|  | | | | |
| **Figure 2 Ntzani *et al*. [8]** | | | | |
| **Dataset** | **Sensitivity** | **Specificity** | **AUC** | **Dataset size** |
| 1 | 80 | 50 | 0.65 | 60 |
| 2 | 25 | 100 | 0.625 | 49 |
| 3 | 40 | 90 | 0.65 | 78 |
| 4 | 40 | 80 | 0.60 | 55 |
| 5 | 75 | 65 | 0.70 | 47 |
| 6 | 40 | 85 | 0.625 | 86 |
| 7 | 55 | 100 | 0.775 | 24 |
| 8 | 10 | 100 | 0.55 | 125 |
| 9 | 70 | 80 | 0.75 | 16 |
| 10 | 80 | 75 | 0.775 | 29 |
| 11 | 55 | 85 | 0.70 | 15 |
| 12 | 90 | 65 | 0.775 | 39 |
| 13 | 55 | 80 | 0.675 | 40 |
| 14 | 40 | 80 | 0.60 | 240 |
| 15 | 70 | 65 | 0.675 | 40 |
| 16 | 100 | 40 | 0.70 | 21 |
| 17 | 80 | 100 | 0.90 | 20 |

| **Table S2.** Average AUC for the gene sets evaluated in the review by Ntzani *et al*. [8] depending on dataset size threshold. | | |
| --- | --- | --- |
|  | | |
| **Figure 1 upper panel Ntzani *et al*. [8]** | | |
| **Threshold dataset size** | **# of datasets included** | **Average AUC** |
| None | 9 | 0.817 |
| 10 | 8 | 0.794 |
| 20 | 7 | 0.786 |
| 30 | 6 | 0.758 |
| 40 | 4 | 0.713 |
| 50 | 4 | 0.713 |
|  | | |
| **Figure 1 lower panel Ntzani *et al*. [8]** | | |
| **Threshold dataset size** | **# of datasets included** | **Average AUC** |
| None | 8 | 0.703 |
| 10 | 7 | 0.679 |
| 20 | 6 | 0.65 |
| 30 | 5 | 0.605 |
| 40 | 5 | 0.605 |
| 50 | 4 | 0.60 |
|  | | |
| **Figure 2 Ntzani *et al*. [8]** | | |
| **Threshold dataset size** | **# of datasets included** | **Average AUC** |
| None | 17 | 0.690 |
| 10 | 17 | 0.690 |
| 20 | 14 | 0.670 |
| 30 | 11 | 0.648 |
| 40 | 8 | 0.625 |
| 50 | 6 | 0.613 |

**References**

[1] Zhao, H, Ljungberg, B, Grankvist, K, Rasmuson, T, Tibshirani, R, Brooks, JD. Gene expression profiling predicts survival in conventional renal cell carcinoma. PLoS Med 2006;3:e13.

[2] Garber, ME, Troyanskaya, OG, Schluens, K, et al. Diversity of gene expression in adenocarcinoma of the lung. Proc Natl Acad Sci U S A 2001;98:13784-13789.

[3] Wang, Y, Klijn, JG, Zhang, Y, et al. Gene-expression profiles to predict distant metastasis of lymph-node-negative primary breast cancer. Lancet 2005;365:671-679.

[4] Miller, LD, Smeds, J, George, J, et al. An expression signature for p53 status in human breast cancer predicts mutation status, transcriptional effects, and patient survival. Proc Natl Acad Sci U S A 2005;102:13550-13555.

[5] Beer, DG, Kardia, SL, Huang, CC, et al. Gene-expression profiles predict survival of patients with lung adenocarcinoma. Nat Med 2002;8:816-824.

[6] Chen, HY, Yu, SL, Chen, CH, et al. A five-gene signature and clinical outcome in non-small-cell lung cancer. N Engl J Med 2007;356:11-20.

[7] van de Vijver, MJ, He, YD, van't Veer, LJ, et al. A gene-expression signature as a predictor of survival in breast cancer. N Engl J Med 2002;347:1999-2009.

[8] Ntzani, EE, Ioannidis, JP. Predictive ability of DNA microarrays for cancer outcomes and correlates: an empirical assessment. Lancet 2003;362:1439-1444.

[9] Zien, A, Fluck, J, Zimmer, R, Lengauer, T. Microarrays: how many do you need? J Comput Biol 2003;10:653-667.

[10] Chang, HY, Sneddon, JB, Alizadeh, AA, et al. Gene expression signature of fibroblast serum response predicts human cancer progression: similarities between tumors and wounds. PLoS Biol 2004;2:E7.

[11] Liu, R, Wang, X, Chen, GY, et al. The prognostic role of a gene signature from tumorigenic breast-cancer cells. N Engl J Med 2007;356:217-226.

[12] Seigneuric, R, Starmans, MH, Fung, G, et al. Impact of supervised gene signatures of early hypoxia on patient survival. Radiother Oncol 2007;83:374-382.
